# Cerebral artery dilation during transient ischemia is impaired by amyloid β deposition around the cerebral artery in Alzheimer’s disease model mice

**DOI:** 10.1186/s12576-020-00785-8

**Published:** 2020-12-10

**Authors:** Nobuhiro Watanabe, Yoshihiro Noda, Taeko Nemoto, Kaori Iimura, Takahiko Shimizu, Harumi Hotta

**Affiliations:** 1 Department of Autonomic Neuroscience, Tokyo Metropolitan Institute of Gerontology, 35-2 Sakaecho, Itabashi-ku, Tokyo, 173-0015 Japan; 2 Animal Facility, Tokyo Metropolitan Institute of Gerontology, Tokyo, 173-0015 Japan; 3 Aging Stress Response Research Project Team, National Center for Geriatrics and Gerontology, Aichi, 474-8511 Japan

**Keywords:** Alzheimer’s disease, Amyloid β, Cerebral artery, Cerebrovascular response, Ischemia-reperfusion, Two-photon imaging

## Abstract

Transient ischemia is an exacerbation factor of Alzheimer’s disease (AD). We aimed to examine the influence of amyloid β (Aβ) deposition around the cerebral (pial) artery in terms of diameter changes in the cerebral artery during transient ischemia in AD model mice (APP^*NL-G-F*^) under urethane anesthesia. Cerebral vasculature and Aβ deposition were examined using two-photon microscopy. Cerebral ischemia was induced by transient occlusion of the unilateral common carotid artery. The diameter of the pial artery was quantitatively measured. In wild-type mice, the diameter of arteries increased during occlusion and returned to their basal diameter after re-opening. In AD model mice, the artery response during occlusion differed depending on Aβ deposition sites. Arterial diameter changes at non-Aβ deposition site were similar to those in wild-type mice, whereas they were significantly smaller at Aβ deposition site. The results suggest that cerebral artery changes during ischemia are impaired by Aβ deposition.

## Background

Alzheimer’s disease (AD) is the most common type of dementia. Based on the amyloid cascade hypothesis [1] that AD is triggered by amyloid β (Aβ) depositing in the brain parenchyma, parenchymal Aβ has been the main topic of research. In contrast, it was reported that the decline in cognitive function was inhibited by controlling vascular risks in humans [2, 3] and that cognitive impairments are accelerated by incorporating vascular risks, such as transient cerebral ischemia and chronic hypoperfusion by occluding carotid arteries, in AD model mice [4–7]. Thus, cerebrovascular dysfunctions have been recently considered to contribute to the pathological mechanism of AD [8, 9].

Neurons in the cerebral cortex and hippocampus, which exhibit degenerative changes in AD, are vulnerable to ischemia [10–14]. Cerebral blood flow (CBF) is decreased by transient occlusion of the carotid artery, leading to the death of cortical and hippocampal neurons. However, the CBF decrease during carotid artery occlusion is mitigated by the systemic administration of nicotine [12] or electrical stimulation of the nucleus basalis of Meynert [11]. Consequently, the number of neuronal death in the cerebral cortex and hippocampus is reduced [10–12]. In response to the occlusion of the unilateral common carotid artery, pial artery initiated to rapidly dilate at the onset of occlusion and maximally dilate approximately 12 s later in anesthetized rats [15]. Such ischemia-induced cerebrovascular change may be a physiological response for securing CBF. In AD model mice, neuronal death following transient cerebral ischemia [6] and the cerebral infarction volume after the middle cerebral artery occlusion [16] are more severe than those in wild-type mice. However, the alteration of cerebrovascular response to ischemia in AD model mice has yet to be elucidated.

Aβ depositions in walls (smooth muscle layer) of cerebrovasculature, including pial artery, were found in more than 78% of autopsy cases of patients with AD [17–22]. In AD model mice, responses of vasodilation by CO_2_ inhalation [23] and vasoconstriction by laser stimulation applied to vascular smooth muscles [24] were attenuated in the cerebrovasculature, where Aβ was deposited compared with vasculature, where Aβ was not deposited. Based on these previous studies, we hypothesized that cerebrovascular response to ischemia was impaired by Aβ deposition in the vasculature. The present study aimed to examine the association between the change in cerebral artery diameter by transient carotid artery occlusion and Aβ deposition around the cerebrovasculature using two-photon imaging.

## Methods

### Animals

Experiments were performed on Aβ precursor protein (APP) knock-in mice (APP^*NL-G-F*^; 16–24 months of age, *n* = 7) and age-matched non-APP knock-in mice (wild-type, *n* = 3) of both genders. This type of APP knock-in mice reportedly exhibits cognitive declines after six months of age [25–27]. All experimental protocols were in accordance with the “Guidelines for proper implementation of animal experiments” established by the Japan Society for the Promotion of Science in 2006 and approved by the animal care and use committee (approval number 18025) and the committee for recombinant DNA experiments (approval number 188) of the Tokyo Metropolitan Institute of Gerontology.

On the day of two-photon imaging, animals were first anesthetized with isoflurane (4%, 2–3 min) and, thereafter, received urethane injection (1.4 g/kg, subcutaneously). Additional urethane (7–12% of initial dose) was administered when necessary. The sufficient depth of anesthesia was evaluated based on the loss of corneal and withdrawal reflexes. The trachea was cannulated, and mice were artificially ventilated (MiniVent Type 845, Harvard Apparatus, MA, USA) [28]. Rectal temperature was maintained at 37.0 °C–37.5 °C under a feedback-regulated temperature control system (BWT-100A, Bio Research Center, Aichi, Japan).

### Common carotid artery occlusion

The left common carotid artery was exposed by bluntly dissecting the sheath around the artery. Special care was administered not to damage the vagus nerve close to the artery. A loose loop was created around the common carotid artery using a silk suture (size, 5–0, Ethicon, NJ, USA). Liquid paraffin oil was applied to the surgical site, including the carotid artery, to prevent tissues from drying. Furthermore, to occlude the common carotid artery, the loop was tightened by pulling the suture for 30 s and subsequently loosened. In the case of multiple trials of occlusion, the interval between arterial occlusions was at least 6 min.

**Fig. 1:**
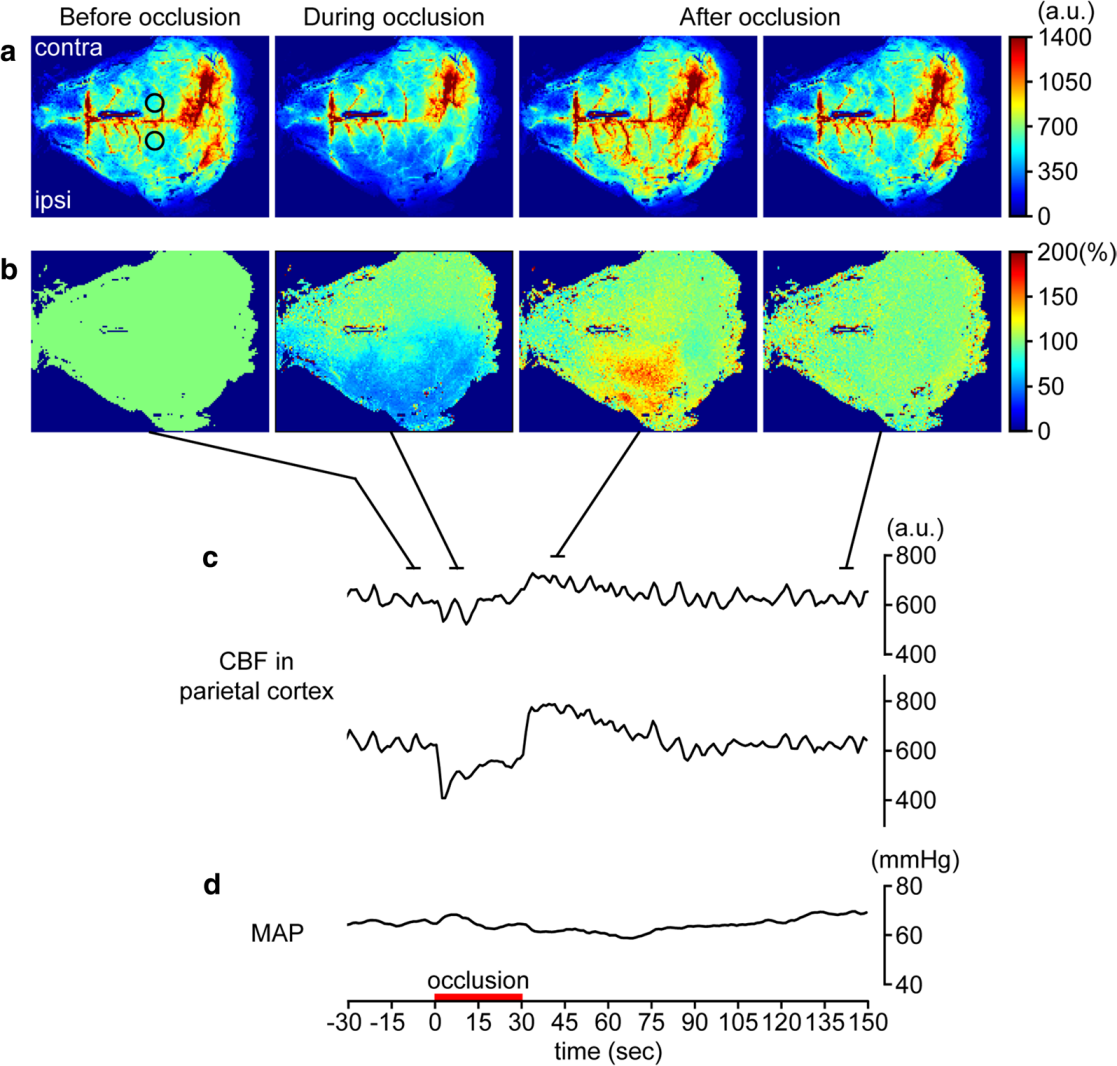
Example data showing the influence of unilateral (left) common carotid artery occlusion on cerebral blood flow (CBF) and mean arterial pressure (MAP). Example images before, during, and after carotid artery occlusion are presented (**a**). Each image is averaged for over 5 s. CBF image is expressed as % change with respect to an image prior to carotid artery occlusion (100%) (**b**). The left and right sides of the image is rostral and caudal sides of the cortex, respectively. Circles in the image before occlusion (**a**) indicate the location of a region of interest (1.5 mm in diameter) to extract CBF values. Time courses of CBF in parietal cortex and MAP changes are presented (**c**, **d**). The upper trace in **c** shows CBF change in the contralateral (right) cortex of carotid artery occlusion, and the lower trace shows change in CBF in the ipsilateral (left) cortex. The period of carotid artery occlusion is indicated by a thick horizontal line on time axis. a.u., arbitrary unit

In preliminary experiments, we examined the influence of unilateral (left) common carotid artery occlusion on CBF. Blood flow in the dorsal surface of the cortex was imaged using a laser speckle flowmeter (moor LFPI2; Moor Instruments, Devon, UK), and arterial pressure was measured via a catheter implanted in the right femoral artery. For CBF measurement, an incision was made into the scalp of the mice while keeping the skull intact. Figure 1 shows that (1) CBF decreases in the ipsilateral cortex to the occlusion side and (2) arterial pressure does not change during unilateral carotid artery occlusion. Based on this result, cerebrovasculature and Aβ in the unilateral cortex were evaluated using two-photon imaging.

### Cranial window preparation for two-photon imaging

On the day prior to imaging, a cranial window was made on the left parietal cortex (approximately 3 mm in diameter; AP, 0 to − 3 mm from Bregma; L, 0.8–3.8 mm from midline). Mice were anesthetized with isoflurane, and each head was fixed to a stereotaxic instrument with ear bars (SR-5M-S, Narishige, Tokyo, Japan). Prior to surgery, fur on the head was trimmed with a conventional clipper, and aseptic techniques were applied. The skin was disinfected using 70% isopropyl alcohol and 10% povidone iodine solution. Anti-inflammatory (carprofen, 5 mg/kg), anti-biotic (cefazolin, 50 mg/kg), and analgesic (buprenorphine, 0.03 mg/kg) drugs were subcutaneously administered before any incisions were made. Procaine hydrochloride was subcutaneously administered to the head for local anesthesia before the scalp was incised.

The skull was partly excised using a dental drill, and the dura mater was kept intact. The excised skull was replaced with a glass slip and fixed on the skull with dental cement [28, 29]. A screw was mounted on the occipital bone to reinforce the bonding of the dental cement. At the end of the surgery, warm saline (0.5 mL) was subcutaneously administered to supplement body fluids.

### In vivo imaging of amyloid β and cerebrovasculature using two-photon microscopy

Aβ and cerebrovasculature were observed using a fluorescence microscope (TCS SP 8 MP; Leica Microsystems, Wetzlar, Germany) through a 25× water-immersion objective lens with correction collar (numerical aperture, 1.00; Leica Microsystems). To evaluate Aβ deposition, Methoxy-X04 (Tocris Bioscience, Bristol, UK), which selectively binds with fibrillar β-sheet deposits and permeates the blood–brain barrier, was utilized [30, 31]. The fluorescent dye was dissolved with dimethyl sulfoxide (DMSO), propylene glycol, and phosphate-buffered saline. Methoxy-X04 solution (0.5% w/v, 1 mL/kg) was intraperitoneally administered a day before the imaging (at the end of surgery). To label blood plasma, FITC-Ficoll (MW ~ 70 kD, Sigma-Aldrich, MO, USA) dissolved in saline (5% w/v, 2 mL/kg) was intraperitoneally administered 10 min before starting the two-photon imaging. The fluorescent dyes administered were excited by a two-photon laser (800–815 nm; Chameleon Vision II, Coherent, CA, USA). The emission signals were detected by external detectors via bandpass filters (460/50 nm for Methoxy-X04 and 520/50 nm for FITC-Ficoll).

For two-photon imaging, we chose the pial arteries running toward the center of parietal cortex from the midline of the cortex, which are branches of the anterior cerebral artery [32]. Among the first to third order branches of the pial artery, two or three locations with a length of > 50 µm without overlying veins (see Additional file 1) were used to evaluate diameter changes in response to the occlusion of the carotid artery. To construct the geometry of cerebrovasculature and Aβ, three-dimensional (3D; XYZ) imaging was executed. One plane (*XY*) image consists of 1024 × 1024 pixels with a pixel size of 0.35 μm. Depth scanning (*Z* axis) was performed with a step size of 2 μm. To examine the vessel diameter change over time in response to carotid artery occlusion, four-dimensional (4D; XYZt) imaging was performed on cerebrovasculature and Aβ (because imaging objects move along the *Z* axis during occlusion), and 3D images were obtained every 15–30 s. The pixel size of one plane (*XY*) image was 0.11–0.52 μm, depending on the zoom factor. Meanwhile, the step size of *Z* axis was 4 μm.

### Data analysis

The obtained images were analyzed offline using the Imaris software (ver. 9.5.0, Bitplane, Belfast, UK). To determine the Aβ deposition, the fluorescence signal of methoxy-X04 was smoothened with minimum of 2 × 2 pixels, and spheres of at least 1 μm diameter were included. For 4D image analyses, the cuboid volume of interests (VOIs) in 10 μm wide were placed across the pial artery after excluding the signal of Aβ, the Gaussian filter was applied with 3 × 3 μm for smoothing, and the image of the artery was binarized by manual adjustment of the threshold intensity. Thereafter, the vessel diameter was measured. Five to twenty VOIs were consecutively placed on an artery, except at vascular bifurcation. The obtained diameters were averaged and treated as a value of each artery.

To examine whether vessel diameter changes during and after carotid artery occlusion, a maximal diameter during the occlusion and a diameter immediately after re-opening the occlusion were compared with the pre-occlusion value using paired *t* test. The vessel diameter before occlusion was compared between wild-type and APP^*NL-G-F*^ mice using the unpaired *t* test.

To assess the association between cerebrovascular response to occlusion and Aβ deposition, subclass analysis was conducted on the pial artery of APP^*NL-G-F*^ mice. VOIs of each artery were classified as presence and absence of Aβ around vasculatures and vessel diameters were separately averaged. Diameter changes during occlusion were expressed as % change with reference to the pre-occlusion value. The diameter change and basal diameter were compared using the unpaired *t* test (between wild-type mice and APP^*NL-G-F*^ mice) or paired *t* test (in APP^*NL-G-F*^ mice between where Aβ deposition presence and absence). The normality of data distribution was confirmed using the Shapiro–Wilk normality test. All statistical analyses were carried out with Prism 6 (GraphPad Software, CA, USA). The calculated p-value was adjusted with Bonferroni correction to account for family-wise type I error for multiple comparisons. Differences with *p* < 0.05 were deemed statistically significant. Data are expressed as median and interquartile range (25–75%).

## Results and discussion

### Two-photon imaging of cerebrovasculature and Aβ deposition

**Fig. 2:**
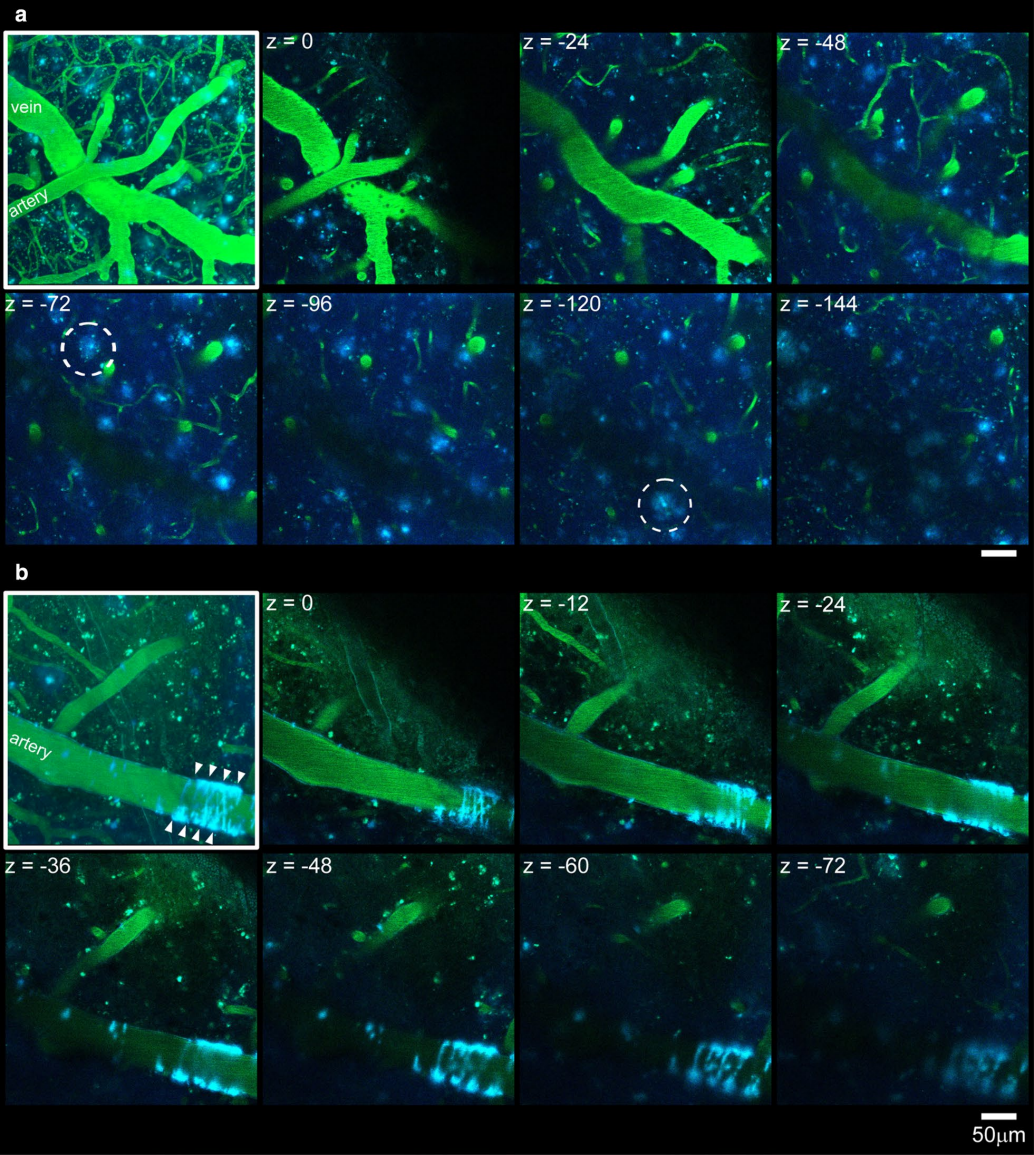
Two-photon image of the left parietal cortex in APP^*NL-G-F*^ mice. Examples of three-dimensional imaging obtained from two individual mice are presented (**a**, **b**). Blood plasma and Aβ are labeled using fluorescent dyes. An image enclosed in a white frame in each panel is a stack image with a maximal intensity projection, and the remaining images are a series of horizontal images. In **a**, a stack image constructed with z-stacks of 320 μm and slice images are presented every 24 μm. In **b**, a stack image constructed with z-stacks of 200 μm and slice images are presented every 12 μm. Deposited Aβ in the cerebral parenchyma is enclosed by a dashed circle, and Aβ deposited around the vasculature is indicated by arrow heads. Scale bar indicates 50 μm

**Fig. 3:**
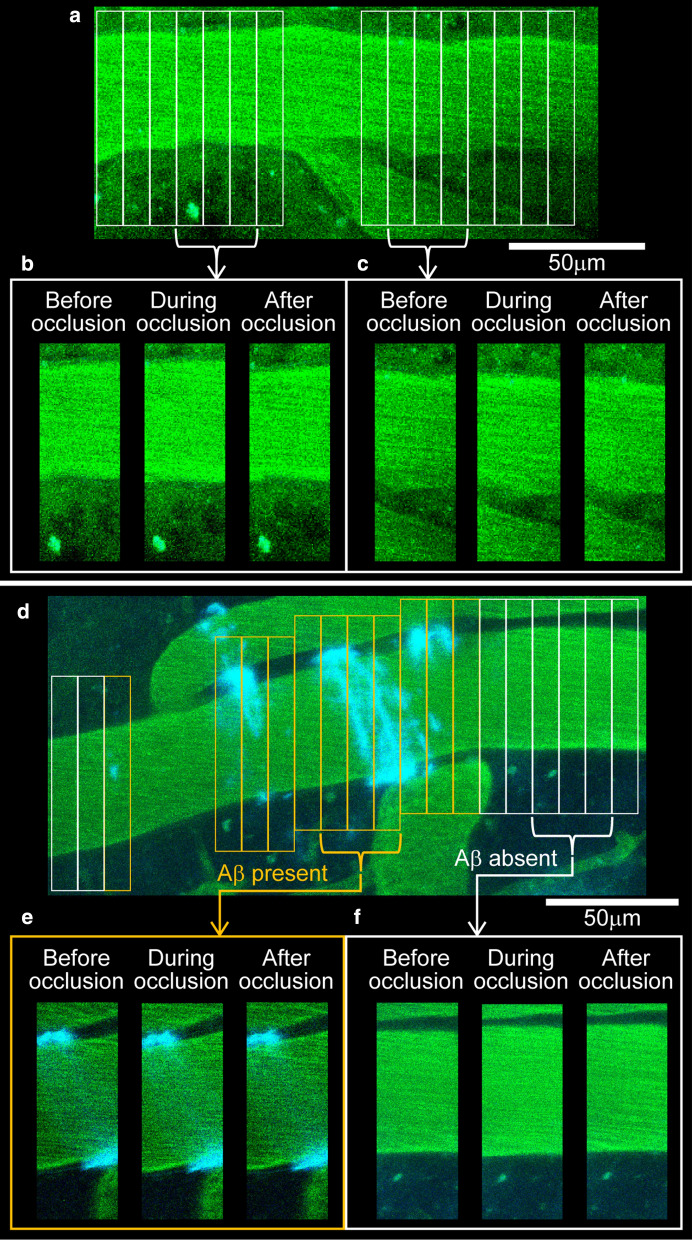
Three-dimensional imaging of pial artery and Aβ obtained before, during and after common carotid artery occlusion. An example image obtained from a wild-type (WT) mouse is illustrated with a maximal intensity projection (**a**). To determine the artery diameter, the pial artery was segmented with a volume of interest (VOI) of 10-μm width (**a**). As an example, three consecutive VOI images from two separated locations before, during, and after carotid artery occlusion are presented (**b**, **c**). An example image of pial artery obtained from an APP^*NL-G-F*^ mouse is presented with a maximum intensity projection (**d**). VOIs on Aβ-present site (orange) and Aβ-absent site (white) are identified. As an example, three consecutive VOI images from Aβ-present site (**e**) and Aβ-absent site (**f**) before, during, and after carotid artery occlusion are presented. Note that slice images are presented in **e** to clarify the diameter change of artery covered with Aβ. Scale bar indicates 50 μm

Figure 2 illustrates a series of horizontal images along the Z axis and its stack image with a maximal intensity projection (enclosed in a white frame) obtained from two APP^*NL-G-F*^ mice. In cerebral parenchyma, Aβ deposited in a spherical shape of approximately 30 μm in diameter was observed (examples are indicated by a dashed circle in Fig. 2a). Additionally, Aβ deposited around a part of the pial artery (Fig. 2b). The Aβ depositions around the artery were found in all seven mice, which underwent two-photon imaging, although the degree of Aβ deposition differed across individuals and arteries. In contrast, such Aβ depositions around the vasculature were not observed in wild-type mice (Fig. 3a).

### Diameter changes of pial artery induced by common carotid artery occlusion

**Fig. 4:**
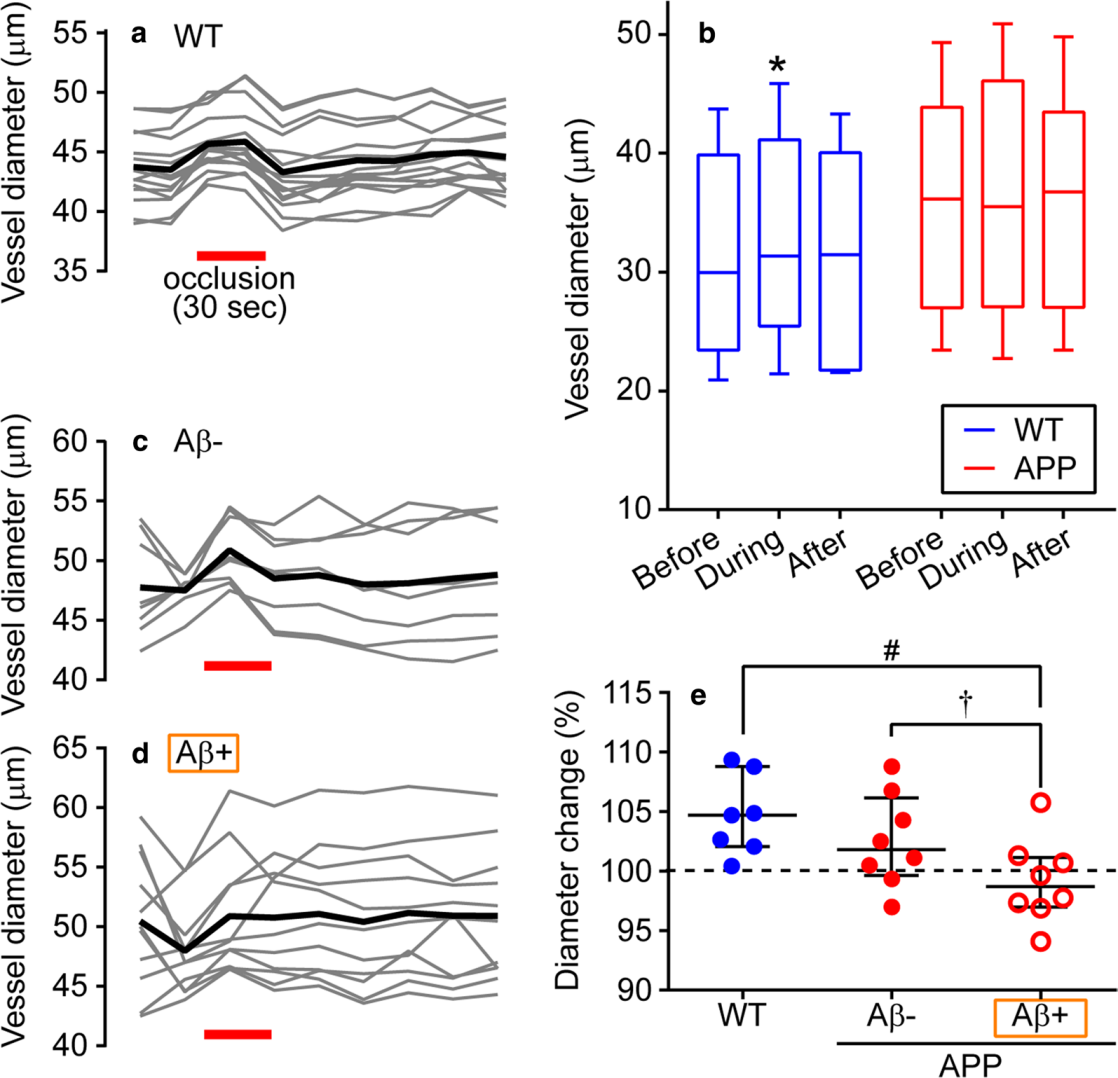
Influence of Aβ deposition around the pial artery on arterial diameter changes in response to common carotid artery occlusion. The time course of artery diameter change in each VOI (thin lines) is shown in Fig. 3a (WT mice), and their average (thick line) is presented (**a**). In this example, a 3D image data were obtained approximately every 16 s. Vessel diameters during carotid artery occlusion and after occlusion re-opening are compared with the diameter before occlusion (**b**). *p < 0.05; significant difference from the value before the occlusion using paired *t* test. The box indicates the median and interquartile range (25%–75%) and the end of whiskers indicates the minimum and maximum values in **b**. **c** and **d** show the artery diameter change time course in each VOI illustrated in Fig. 3d from Aβ-absent (Aβ−) site and Aβ-present (Aβ+) site, respectively. In this example, a 3D image data were obtained approximately every 20 s. Diameter changes during carotid artery occlusion are compared (**e**). Each circle in **e** indicates data of individual artery. ^#^*p* < 0.05; significant difference using unpaired *t* test. ^†^*p* < 0.05; significant difference using paired *t* test. Data are expressed as the median and interquartile range (25–75%) in **e**

In three wild-type mice (a total of 104 VOIs were placed on seven pial arteries), diameter changes of pial artery during and after carotid artery occlusion were evaluated. VOIs were placed on a pial artery except for vascular bifurcation (Fig. 3a). An example image (one pial artery) of a wild-type mouse is shown (Fig. 3a–c). The diameter of the pial artery was stable prior to the unilateral common carotid artery occlusion (Fig. 4a). The artery diameter increased within 16 s following the onset of the occlusion, and the increase was maintained during a 30-s occlusion. The diameter returned to the pre-occlusion level immediately after the occlusion was terminated and slightly increased afterwards. Values of each VOI obtained from one artery exhibited similar trends (Fig. 4a), and responses of the other six arteries in wild-type mice were also similar. Values obtained by VOI analysis on each artery were averaged, and the diameter change over time was assessed on seven arteries. Paired *t* test showed that the diameter of the pial artery (30.0 μm [23.4–39.9 μm]) significantly increased during the common carotid artery occlusion (31.4 μm [25.5–41.1 μm], *p* = 0.030) and the increase became insignificant after the occlusion was terminated (31.5 μm [21.7–40.1 μm], *p* > 0.99) in wild-type mice (Fig. 4b). Such blood vessel diameter responses during the occlusion in wild-type mice were similar to those reported in rats [15]. Due to a considerably rapid response of the cerebrovasculature, the vasodilation apparently resulted from a vascular smooth muscle-derived (myogenic) response to a decrease in perfusion pressure rather than accumulation of metabolites in the brain during ischemia.

Unilateral common carotid artery occlusion was performed on four APP^*NL-G-F*^ mice (a total of 120 VOIs were placed on eight pial arteries). On the pial artery of APP^*NL-G-F*^ mice, there were locations, where Aβ was present and where Aβ was absent (Fig. 3d). Of 120 VOIs, Aβ was present in 54 VOIs (45.0%) and absent in 66 VOIs (55.0%). Regardless of the presence or absence of Aβ, all values obtained by VOI analysis of each artery were averaged, and gross changes in artery diameter during and after carotid artery occlusion were evaluated. In APP^*NL-G-F*^ mice, paired *t* test showed that the diameter of the pial artery (36.2 μm [27.0–43.9 μm]) did not change during (35.5 μm [27.1–46.1 μm], *p* > 0.99) or after (36.7 μm [27.1–43.5 μm], *p* = 0.80) the occlusion (Fig. 4b). The pre-occlusion diameter of the pial artery was not statistically different between wild-type and APP^*NL-G-F*^ mice (*p* = 0.44), consistently with a previous report [23]. The present study is the first report on blood vessel dilation during carotid artery occlusion attenuated in AD model mice. The attenuation of the vascular response during ischemia in AD model mice demonstrated by the present study may be related to reports that neurons of the brain in AD model mice is more vulnerable to transient cerebral ischemia compared with wild-type mice [6, 16]. The present study result may also involve a mechanism, where autoregulation function of cerebrovasculature diminished in patients with AD [33, 34], in the elderly with high Aβ deposition (measured with ^18^F-florbetaben-PET) [35], and in AD model mice [36].

Furthermore, by classifying VOIs of APP^*NL-G-F*^ mice based on the presence and absence of Aβ around the pial artery, an association between diameter changes of pial artery during carotid artery occlusion and Aβ deposition around the vasculature was examined. Sample images illustrate that the artery diameter, where Aβ is absent increases during occlusion (Figs. 3f, 4c), whereas the artery diameter where Aβ was present minimally changed (Figs. 3e, 4d). On each of the eight pial arteries, maximal diameter changes during occlusion, where Aβ was present and absent were averaged separately and compared as the subclass analysis. The paired *t* test revealed that the pre-occlusion diameter was not different between Aβ absent and present sites (36.1 μm [26.6–45.2 μm] vs. 35.5 μm [26.7–42.5 μm], respectively, *p* = 0.99). In contrast, vessel diameter changes during occlusion were significantly smaller at a location, where Aβ was present than that where Aβ was absent (98.7% [97.0%–101.2%] vs. 101.8% [99.6%–106.1%], *p* = 0.017; Fig. 4e). Comparing these responses with that of wild-type mice (104.7% [102.1–108.8%]), the diameter change, where Aβ was present was significantly smaller (*p* = 0.026); however, the response where Aβ was absent was not different (*p* = 0.83). Therefore, the present results denoted that artery dilation during carotid artery occlusion was attenuated at locations where Aβ deposited. Consistent with previous studies that Aβ around blood vessels impairs the response of cerebral blood vessels to CO_2_ inhalation [23] and laser stimulation [24], results of the present study strongly suggest that Aβ may impede various physiological functions of cerebrovasculature.

After euthanasia, the parietal cortex was imaged in two APP^*NL-G-F*^ mice. We observed that the pial artery diameter where Aβ was absent was smaller than that where Aβ was present (Additional file 2b, d). In contrast, when the parietal cortex was imaged in mice before euthanasia, the diameter of pial arteries was similar not only at sites where Aβ was present but also at those where Aβ was absent (Additional file 2a, c). Aβ was reported to be deposited in the tunica media of blood vessel walls (i.e., the smooth muscle layer) [20, 23, 37–39]. Therefore, our observation may indicate that Aβ deposition around the cerebrovasculature acts as a mechanical barrier and restricts vascular diameter changes.

In the present study, fibrillary Aβ deposition was imaged, and the influence of such Aβ on cerebrovascular response to ischemia was examined. Soluble Aβ decreases endothelial nitric oxide synthase [40] and enhances the vasoconstrictive effect of endothelin [41]; however, the imaging method used in the present study could not image soluble Aβ. To clarify the possibility that chemical effects of soluble Aβ attenuate vascular response at the early stage of AD or before Aβ deposits, future studies with earlier stage of AD model animals and other imaging methods are necessary.

## Conclusions

The present study suggests that Aβ depositing around cerebrovasculature attenuated vascular dilation in response to transient ischemia in AD model mice, which is possibly because the diameter change of cerebrovasculature was mechanically impeded.

### Supplementary information

**Additional file 1.** Pial artery geometry and imaging sites. The schema of pial artery derived from the anterior cerebral artery of individual mice is illustrated. A pial artery emerging from the midline border of cranial window was defined as the first order branch. Up to third order branches were evaluated in the study. Pial artery data from seven locations in three WT mice and eight locations in four APP mice were obtained in total, and the location evaluated was indicated using numbers (2–3 locations per mice). The branches evaluated were 2 first order and 5 second order branches in WT mice as well as 1 first order, 5 second order, and 2 third order branches in APP mice. The branch order distribution was not statistically different between WT and APP mice (chi-square test; *p* = 0.32).

**Additional file 2.** A comparison of vascular diameter before and after euthanasia between Aβ-present and Aβ-absent sites. Example images of cerebrovasculature and Aβ obtained from an APP^*NL-G-F*^ mouse are illustrated with a maximal intensity projection. The same location of parietal cortex was imaged before (a, c) and after (b, d) euthanasia. Stack images in panels (a, b) were constructed with z-stacks of maximum 172 μm and those in panels (c, d) were constructed with z-stacks of 290 μm. The pial artery at the locations where Aβ deposition is present is indicated by orange-colored arrow heads and locations where Aβ deposition is absent is indicated by white-colored arrow heads. Pial arteries are generally shrunk after euthanasia, and the diameter of the artery is narrower where Aβ deposition is absent. Scale bar indicates 50 μm.

## Data Availability

The datasets used and analyzed during the current study are available from the corresponding author on reasonable request.
