# Supplementary figures and images for: Cerebral artery dilation during transient ischemia is impaired by amyloid β deposition around the cerebral artery in Alzheimer’s disease model mice
Source: J Physiol Sci. 2020 Dec 10;70:57. doi: 10.1186/s12576-020-00785-8 (PMC10718030; doi:10.1186/s12576-020-00785-8)

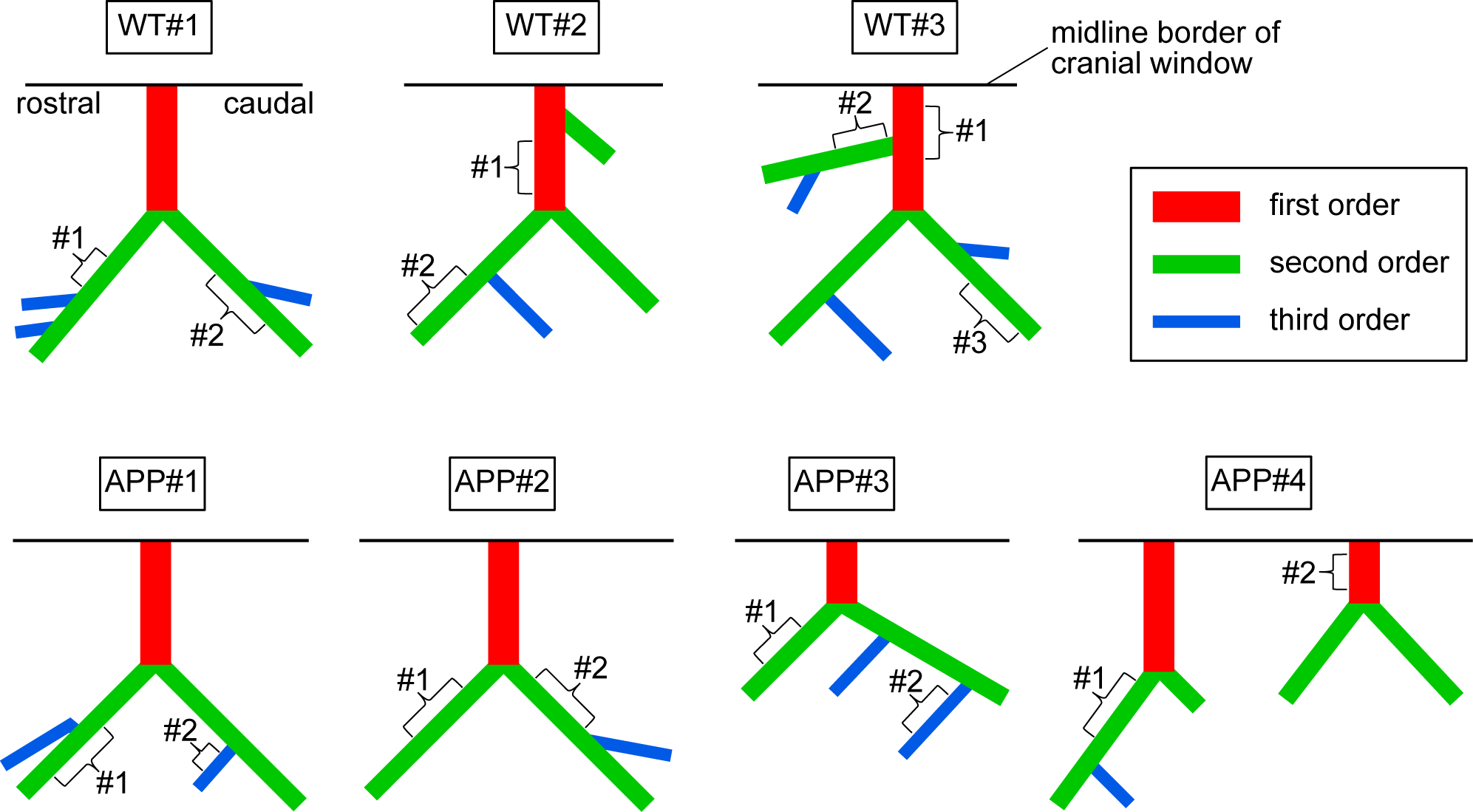

Supplement: Supplementary file 1 — Additional file 1. Pial artery geometry and imaging sites. The schema of pial artery derived from the anterior cerebral artery of individual mice is illustrated. A pial artery emerging from the midline border of cranial window was defined as the first order branch. Up to third order branches were evaluated in the study. Pial artery data from seven locations in three WT mice and eight locations in four APP mice were obtained in total, and the location evaluated was indicated using numbers (2–3 locations per mice). The branches evaluated were 2 first order and 5 second order branches in WT mice as well as 1 first order, 5 second order, and 2 third order branches in APP mice. The branch order distribution was not statistically different between WT and APP mice (chi-square test; p = 0.32). [file 12576_2020_785_MOESM1_ESM.tif]

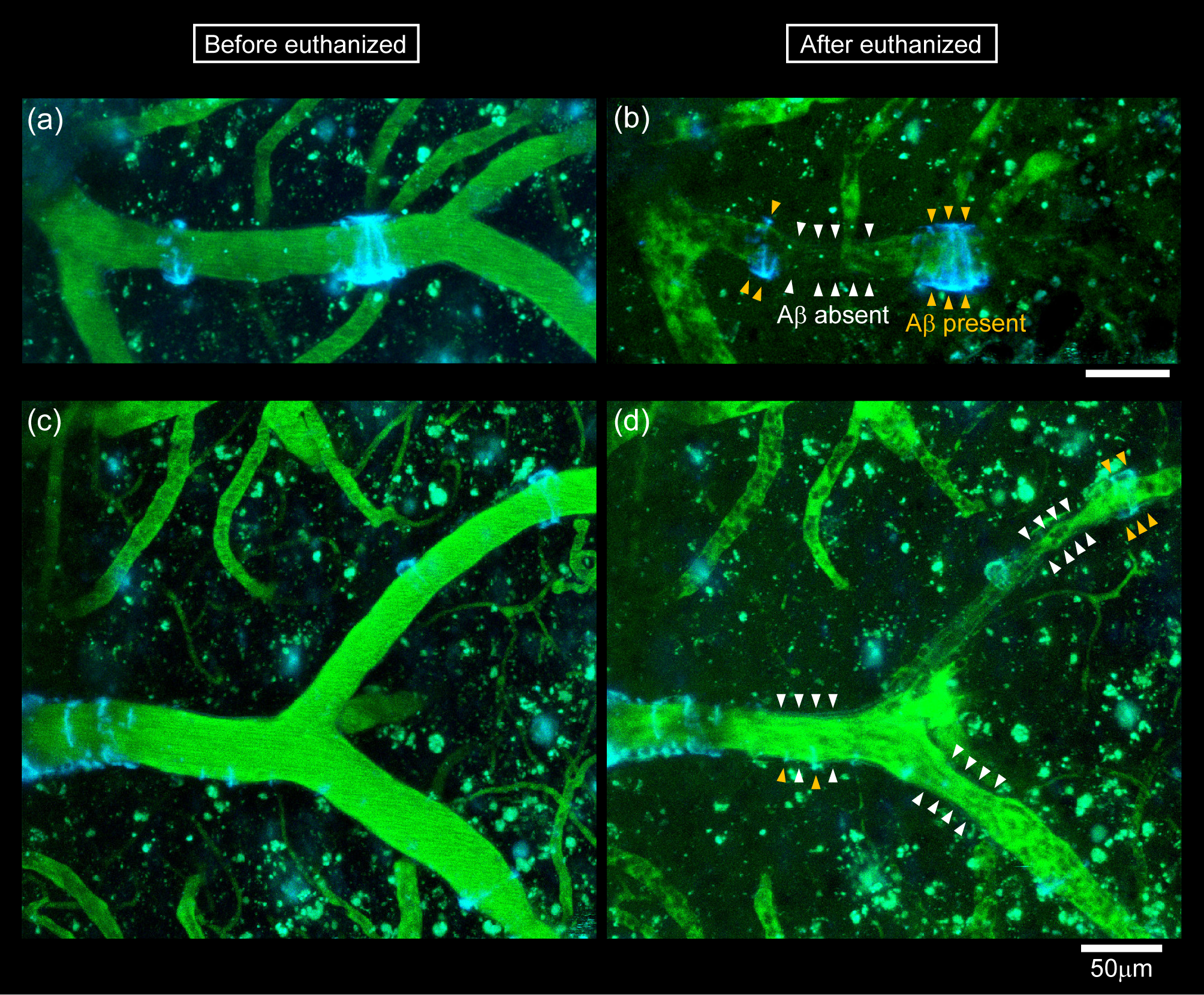

Supplement: Supplementary file 2 — Additional file 2. A comparison of vascular diameter before and after euthanasia between Aβ-present and Aβ-absent sites. Example images of cerebrovasculature and Aβ obtained from an APPNL-G-F mouse are illustrated with a maximal intensity projection. The same location of parietal cortex was imaged before (a, c) and after (b, d) euthanasia. Stack images in panels (a, b) were constructed with z-stacks of maximum 172 μm and those in panels (c, d) were constructed with z-stacks of 290 μm. The pial artery at the locations where Aβ deposition is present is indicated by orange-colored arrow heads and locations where Aβ deposition is absent is indicated by white-colored arrow heads. Pial arteries are generally shrunk after euthanasia, and the diameter of the artery is narrower where Aβ deposition is absent. Scale bar indicates 50 μm. [file 12576_2020_785_MOESM2_ESM.tif]
